# Detection of acute ventilatory problems via magnetic induction in a newborn animal model

**DOI:** 10.1038/s41390-021-01594-4

**Published:** 2021-06-08

**Authors:** Sabrina C. Behr, Christopher Platen, Pascal Vetter, Nicole Heussen, Steffen Leonhardt, Thorsten Orlikowsky, Konrad Heimann

**Affiliations:** 1 Department of Neonatology, University Children’s Hospital, RWTH Aachen University, Aachen, Germany; 2 Philips Chair for Medical Information Technology, RWTH Aachen University, Aachen, Germany; 3 Department of Medical Statistics, Medical Faculty RWTH Aachen University, Aachen, Germany; 4 Center of Biostatistics and Epidemiology, Medical School, Sigmund Freud University, Vienna, Austria

## Abstract

**Background:**

Magnetic induction measurement (MIM) is a noninvasive method for the contactless registration of respiration in newborn piglets by using measurement coils positioned at the bottom of an incubator. Acute pulmonary problems may be determinants of poor neurological and psychomotor outcomes in preterm infants. The current study tested the detection of pulmonary ventilation disorders via MIM in 11 newborn piglets.

**Methods:**

Six measurement coils determined changes in magnetic induction, depending on the ventilation of the lung, in comparison with flow resistance. Contactless registration of induced acute pulmonary ventilation disorders (apnea, atelectasis, pneumothorax, and aspiration) was detected by MIM.

**Results:**

All pathologies except aspiration were detected by MIM. Significant changes occurred after induction of apnea (three coils), malposition of the tube (one coil), and pneumothorax (three coils) (*p* ≤ 0.05). No significant changes occurred after induction of aspiration (*p* = 0.12).

**Conclusions:**

MIM seems to have some potential to detect acute ventilation disorders in newborn piglets. The location of the measurement coil related to the animal’s position plays a critical role in this process. In addition to an early detection of acute pulmonary problems, potential information pointing to a therapeutic intervention, for example, inhalations or medical respiratory analepsis, may be conceivable with MIM in the future.

**Impact:**

- MIM seems to be a method in which noncontact ventilation disorders of premature and mature infants can be detected.
- This study is an extension of the experimental setup to obtain preliminary evidence for detection of respiratory activity in neonatal piglets. For the first time, MIM is used to register acute ventilation problems of neonates.
- The possibility of an early detection of acute ventilation problems via MIM may provide an opportunity to receive patient-side information for therapeutical interventions like inhalations or medical respiratory analepsis.

## Introduction

Apneas or disorders of the respiratory system, such as pneumothorax or tube shifts, are clinically detected by alarm-triggering respirators, pulse oximeters, and heart rate monitors. Regarding ventilation, boundary values can be set for respiratory pressure and gas flow, and oxygen saturation, which when exceeded, can indicate the need for intervention. As such, alarms can be activated by a variety of different causes; the availability of more detailed information to identify these causes is likely to enhance clinical effectiveness on a real-time basis. A drop in oxygen saturation below the lower target usually has causes that begin earlier than current measurement methods can detect.

Thus, it would be desirable to have a noninvasive monitoring method that permits real-time evaluations of regional lung function, thereby reducing the often high number of X-ray images that are required due to a lack of imaging alternatives.

Noncontact methods would be of particular interest to protect the sensitive skin and reduce the probability of nosocomial infections because this would avoid skin–electrode contact. In fact, techniques for noncontact vital sign monitoring have recently gained a lot of attention for various applications.^1,2^

Capacitive coupling, for example, to replace the cable-bound electrocardiogram (ECG) on the neonatal intensive care unit (NICU) has been investigated.^3^ Recent reviews focus particularly on neonatal heart rate monitoring.^4,5^ Regarding the respiratory rate, infrared thermography has demonstrated the potential for application as it allows to monitor not only the breathing rate of the neonate but also the surface temperature distribution.^6^

Another method offering such noninvasive, contactless monitoring is the so-called “magnetic induction measurement” (MIM). This is a method that allows the contactless, noninvasive detection of impedance changes in biological tissue via the short-term alternation of magnetic fields.

Different studies have investigated the applicability of MIM in a range of medical fields, including the detection of cerebral edema^7^ or elevated iron levels in the liver,^8^ as well as the monitoring of cardiac^9^ and respiratory activity.^10^ Such investigations have revealed a correlation between pulmonary gas volume and MIM, from which the respiratory rate and tidal volume can be calculated.

Apnea detection in adults using MIM is also possible: The MIM is a flow sensor and zero flow is visible even if the MIM signal only gives artificial units.^11,12^

It is notable that, in addition to single-channel MIM systems, studies have also used multichannel systems to improve the recording of regional information in the future.^13–19^ In line with this, the Department of Medical Information Technology of the RWTH Aachen has developed a MIM setup, including image reconstruction with a multichannel system, for the simultaneous monitoring of heart and lung function.^20,21^ This setup has been successfully used to obtain preliminary evidence for the detection of respiratory activity in neonatal piglets via MIM.^22^

Therefore, our aim was to test the hypothesis that an extension of the established newborn animal model due to induced pathologies (apnea, tube malposition, pneumothorax, and aspiration) is possible, and these pathologies can be registered by standard monitoring (pulse oximetry, ECG, and X-ray) and MIM.

## Animals, materials and methods

### MIM (system for data collection)

**Fig. 1:**
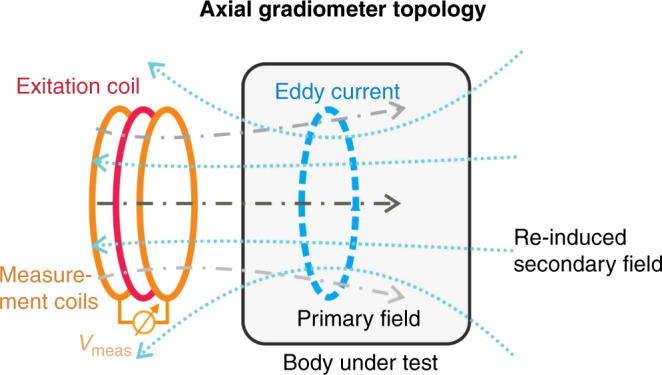
Axial gradiometer topology. Principle of axial gradiometer-based MIM, modified from ref. ^2^.

The MIM uses the principle of electromagnetic induction, which induces eddy currents flowing on closed circular trajectories in the conductive material. Regarding any charge flow, MIM induces a secondary field that can be measured in a sensing coil arrangement (in axial gradiometers; this is a differential topology to compensate for the common-mode fraction resulting from the primary field) from outside the body (Fig. 1).

Air-filled lung tissue is a poor electrical conductor (with a high specific electrical resistance of ~10–20 Ωm during inspiration and expiration), while blood-filled tissue conducts much better (with a fairly low specific electrical resistance coating of ~1.6 Ωm). Ventilation disorders induce variable changes in the distribution of impedance. These changes can be detected by measuring the corresponding eddy current induction. This is based on different characteristics in resistance, for example, after the induction of pathologies.

Variations in air and fluid content (due to local ventilation and perfusion, but also due to infection [e.g., pus], atelectasis, or pneumothorax) change the specific tissue resistance, which is reflected in modulations of the secondary field and can be observed from outside. Various topologies come to mind for possible gradiometer coil arrangements and have been investigated in the past, including the axial arrangement used in this research paper. We note that, in addition to gradiometer coil arrangements, there is also the option of using a single coil arrangement operating in an oscillator mode.^23,24^

Coils can also be combined to form arrays, as performed in this work. While combining coils is difficult in oscillatory coil operation due to crosstalk, it adds spatial resolution for gradiometer coil arrays. Therefore, such gradiometer arrangements are recommended if spatial resolution is desired.

### Animal model

All animal experiments were conducted in accordance with section 1, paragraph 8 of the Protection of Animals Act after approval from the provincial government of North Rhine-Westphalia, Germany, as a final experiment (file number: 84-02.04.2015.A323). Newborn piglets at 24 to 48 h of age were chosen as experimental animals because they share similar basic proportions to human newborns. Therefore, specific procedures of daily routine care could be simulated and also specific pathologies (e.g., pneumothorax). According to an identical test procedure (Table 1), animal experiments were conducted at the Department of Veterinary Medicine of the University Hospital of Aachen. The animals were given total intravenous analgosedation with midazolame and ketamine and received parenteral dextrose infusion (Table 1), to avoid a catabolic metabolism, throughout the experiment. Consequently, the experimental setup imitated the general practice in premature and mature infants, including blood gas analysis that allows an early identification of acidosis, possibly due to a loss of volume.

**Table 1:** Experimental setup.

1. Induction of inhalation anesthesia with 2% isoflurane
2. Placement of intravenous access at one ear
3. Intravenous sedation with midazolame 0.1 mg/kg. Continuous infusion with ketamine (1 ml + 9 ml NaCl 0.9%): 0.6 ml/h → 6 mg/h, midazolame (purely) 0.6 ml/h → 3 mg/h. Monitoring using ECG and pulse oximetry at all four extremities
4. Placement of second venous access at the other ear
5. Intravenous parenteral dextrose infusion with Pädiafusin® I/Ringer®-Lsg. (Baxter, Unterschleißheim, Germany). Premedication for intubation with pentobarbital (1 ml + 15 ml NaCl 0.9%) 1 ml → 10 mg intravenously, fentanyl (1 ml + 9 ml NaCl) 2 ml → 10 mg intravenously
6. Intubation with 3.5 mm endotracheal tube with surfactant channel up to tag 11
7. Initial ventilation via manual ventilation bag, then with respirator (frequency 40, p max 15, flow 8, PEEP 5), later p max 10 over 60 min
8. Vital parameter measurement: conventional via ECG and pulse oximetry and via MIM. Measurement of arterial access with subsequent BGA monitoring of respiratory settings. Blood pressure monitoring via arterial access
9. Malposition of endotracheal tube: ventilation of animal is stopped five times each for 1 min, while vital parameters are measured both conventionally and via MIM
10. Atelectasis: the tube is introduced into the right lung → position check via X-ray. Synchronously, vital parameters are measured conventionally and by MIM in different positions. Thereafter, the “reopening” of the lung is documented via electrical impedance tomography if possible
11. Pneumothorax: placement of an intrapleural catheter, position check via X-ray. Then, intrapleural insufflation of 20 ml of air → radiological check of the degree of pneumothorax
12. Aspiration: 20 ml of NaCl 0.9% is applied intrapleurally and 10 ml is applied intrapulmonarily via surfactant channel
13. Finalization of the animal experiment with 70 mg of pentobarbital intravenously
During monitoring, as required: Bolus midazolame = 0.5 ml ≙ 0.5 mg Bolus ketamine = 0.4 ml ≙ 4 mg Bolus fentanyl = 2 ml ≙ 10 µg Bolus pentobarbital = 1 ml ≙ 10 mg
Continuous infusion: 10 ml of ketamine in 50 ml (concentration 10 mg/ml); 10 ml midazolame in 50 ml (concentration 5 mg/ml)
Bolus administration: • 20 ml syringe midazolame (concentration 1 mg/ml); 20 ml syringe ketamine (concentration 10 mg/ml) • 20 ml syringe fentanyl (concentration 5 µg/ml) • 20 ml syringe pentobarbital (concentration 10 mg/ml) • Midazolame (Dormicum®) (pure) 5 mg/ml® 1 ml + 4 ml NaCl 0.9% for 1 mg/ml • Ketamine (pure) 100 mg/ml® 1 ml + 9 ml NaCl 0.9% for 10 mg/ml • Fentanyl (pure) 50 µg/ml® 1 ml + 9 ml NaCl 0.9% for 5 µg/ml • Pentobarbital (Narcoren®) (pure) 160 mg/ml; 1 ml + 15 ml NaCl 0.9% for 10 mg/ml

### Measurements

**Fig. 2:**
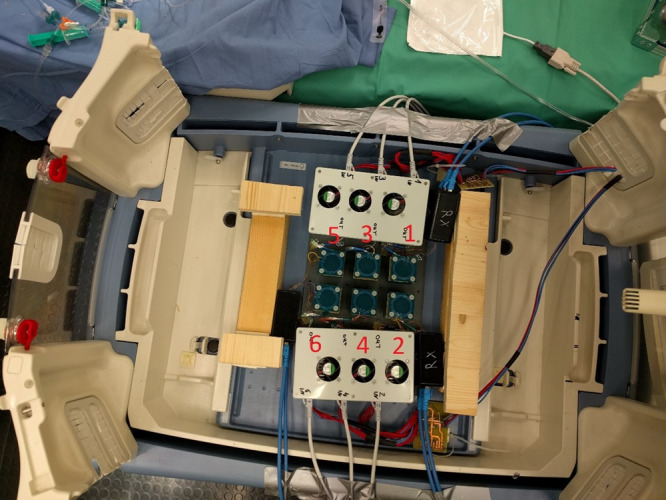
Experimental incubator. Setup of the measurement system at the bottom of the experimental incubator (coils 1–6).

The incubator “Caleo” (Dräger Medical GmbH, Lübeck, Germany) contained six combined measuring and excitation coils, each with a diameter of ~4 cm, with the possibility of measuring with up to 12 channels at six excitation frequencies. The coils were positioned at the bottom of the incubator below the surface of the animal at a distance of ~3 cm from the body and calibrated to the excitation frequency of 10 MHz (Fig. 2).

The reduction, filtration, and detention of the measurement signals occurred using a self-contained demodulation software.^20^ These signals were visualized subsequently online utilizing another software. Both software packages were developed at the RWTH of Aachen.^20^

The reference signal that was generated via pulse oximetry (IntelliVue MP70 Neonatal, Philips, Eindhoven, The Netherlands) and an air flow gauge (flow resistance and pressure differential gauge) was analyzed in the same way.

The setup of the animal model established was maintained and changed from different ventilation modes (continuous positive airway pressure, high-frequency oscillatory ventilation)^22^ to four pathologies under conventional ventilation. The following pathologies were induced: apnea, atelectasis with tube malposition, pneumothorax, and aspiration.

It was guaranteed that each animal was positioned in an equal position to the measurement coils by using a tailored foam pad. The ventilation of the animals was recorded by MIM, and the flow resistance over a minimum period of 10 min was recorded before the induction of each pathology to guarantee an interference-free signal on a high-quality level during the whole experimental setup. All the air flow measurements recorded by the respirator were compared directly to the MIM signal data. After the induction of each pathology except aspiration, chest X-ray was performed for validation.

The parameters of vital functions and ventilation disorders were monitored and recorded using ECG electrodes (heart rate), oxygen saturation measurements (pulse oximetry), capnometry, pulmonary mechanics via the respirator, radiological tools (X-ray unit of the Institute of Experimental Animal Science), and clinically by auscultation.

First, apnea was induced five times (for 1 min each time) under analgosedation and, if necessary, relaxation by disconnecting the animal from the respirator. Atelectasis was induced after positioning the tube into the right main bronchus to induce a unilateral ventilation of the lung.

**Fig. 3:**
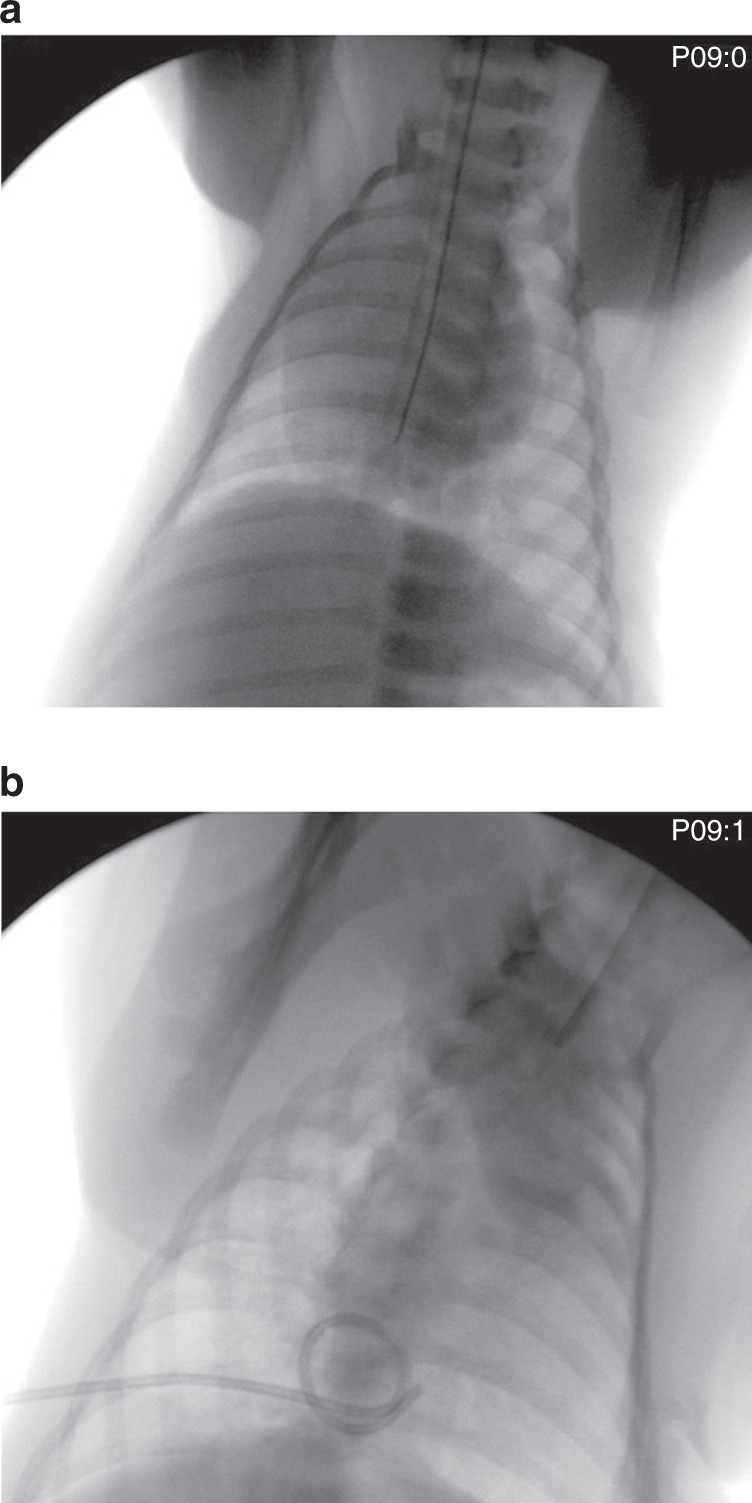
X-ray. Chest X-ray of an experimental animal (**a**) tube malposition and (**b**) pneumothorax.

The tube’s position was controlled by chest X-ray (Fig. 3a). This resulted in a pronounced ventilation disturbance in the left lung, causing the formation of atelectasis, simultaneous hyperinflation of the right lung, and overall changes in the lung mechanics. In the next step, a maximum of 20 ml of air was insufflated into the right half of the thorax via an intrapleural catheter, causing pneumothorax (Fig. 3b).

Finally, aspiration was simulated by the instillation of an isotonic saline solution (NaCl 0.9%) via the surfactant channel of the tube. Afterward, the animals were euthanized with a high dose of pentobarbital.

### Statistical analysis

In order to compare measurements between each of the four pathologies to the reference signal, a linear mixed-effects model with a random intercept and unstructured covariance matrix was fitted to the data to account for repeated measurements within animals. The type of pathology (or reference) and coil were considered as main effects. In addition, a term to identify the interaction between type and coil was included in the model to specify the linear contrast for the comparisons intended. Model assumptions and fit were checked by visual inspection of the residuals and influence diagnostics. Missing values were considered by a likelihood-based approach within the framework of mixed linear models with the assumption that missing values will occur at random. The significance level was set at 5% for all comparisons. No adjustment to the significance level was made due to the explorative nature of the evaluation. Results were reported as means ± standard deviations (SD) and two-sided *p* values. All analyses were performed with SAS version 9.4 (PROC MIXED) (SAS Institute Inc., Cary, NC).

## Results

The current study examined 11 newborn piglets, aged 24–48 h with a weight range of 2220–2559 g. This corresponds approximately to premature infants at a gestational age of 36 weeks.

### Vital and ventilation parameters

The mean and standard deviation for the vital parameters before, during, and after induction of apnea 1–5, tube malposition, pneumothorax, and aspiration are shown in Table 2.

**Table 2:** Data (mean, SD) of heart rate and oxygen saturation of the experimental animals after induction of apnea 1–5, tube malposition, pneumothorax, and aspiration.

Variable	Apnea 1		Apnea 2		Apnea 3		Apnea 4		Apnea 5		Tube malf.		Pneumothorax		Aspiration	
	Mean	SD	Mean	SD	Mean	SD	Mean	SD	Mean	SD	Mean	SD	Mean	SD	Mean	SD
Heart rate	125.2	21.3	119.0	20.4	125.1	31.0	116.2	22.5	105.5	18.0	101.7	17.3	100.1	21.1	105.0	31.7
Oxygen saturation	98.3	3.7	97.7	2.9	98.7	2.9	98.5	3.3	97.9	3.7	99.0	2.6	69.4	36.2	49.7	39.7

The induction of apnea caused a change in the heart rate up to 23 beats/min (20%), while the oxygen saturation remained stable (max. change 3%). A verification with a chest X-ray was not necessary because of the clinical absence of thoracic excursions. By contrast, the tube malposition in the right main bronchus was checked in all 11 piglets via chest X-ray (Fig. 3a). No clinically evident change in heart rate or oxygen saturation occurred, similar to during the daily routine of premature infants. Pneumothorax was also verified with X-ray after a puncture of the right pleura of the animals (Fig. 3b). A pneumothorax could be induced after puncture and insufflation of a maximum of 20 ml of air in all animals. An obvious change of heart rate could not be detected, while oxygen saturation changed up to 36%. Induction of aspiration showed a high change in both heart rate (31%) and oxygen saturation (40%). A radiological control was not performed.

### MIM

**Fig. 4:**
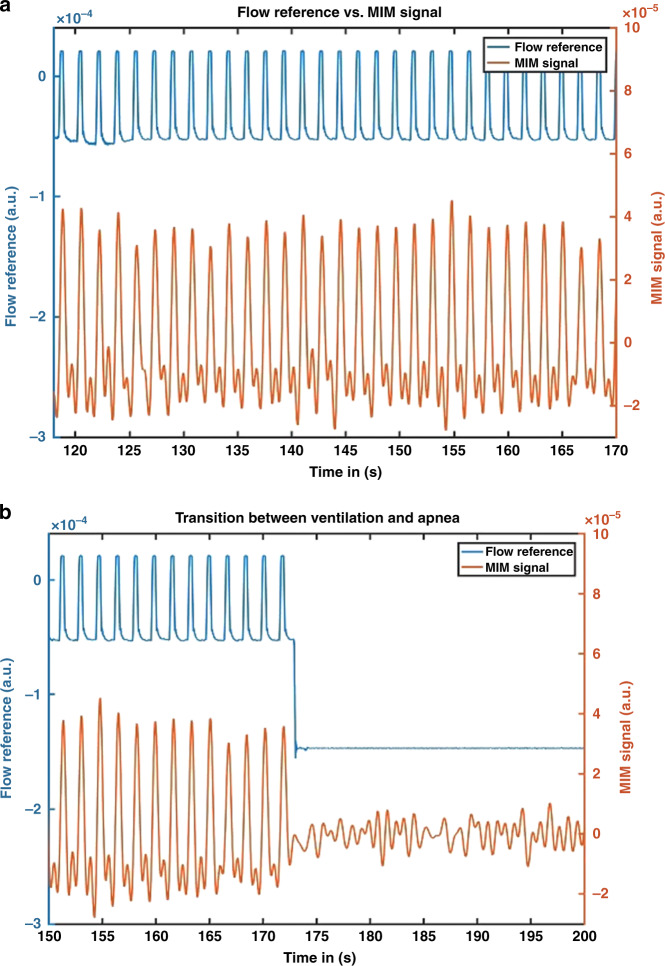
Flow reference and MIM signal. Comparison of a reference signal (blue) and MIM (red) during wash-out time (**a**) and after induction of an apnea (**b**).

The ventilation of the animals was recorded by MIM, and a reference signal over a wash-out time of 10 min was recorded before the induction of each pathology to guarantee an interference-free signal on a high-quality level (Fig. 4a). A significant change in the level of the amplitude was defined as an effective registration of a pathology via MIM. This occurred after the induction of all five apnea events (*p* ≤ 0.05; Table 3 and Fig. 4a). Regarding tube malposition, a significant difference before and after positioning the tube in the right main bronchus was observed (*p* ≤ 0.05; Table 3). Similarly, a significant change in the level of the amplitude (*p* ≤ 0.05; Table 3) was registered after puncture of the right lung and insufflation of a maximum of 20 ml of air (pneumothorax). However, no significant difference between the measurement of the reference signal and MIM before and after induction of the pathology (*p* = 0.75; Table 3) occurred during aspiration.

**Table 3:** *P* value from the comparison of the six measurement coils after induction of pathology (apnea 1–5, tube malposition, pneumothorax, aspiration) to the reference measurement/signal.

Pathology	Coil 1	Coil 2	Coil 3	Coil 4	Coil 5	Coil 6
	*p* value	*p* value	*p* value	*p* value	*p* value	*p* value
Apnea 1	0.2861	0.0975	0.0532	0.1432	**<0.05**	**<0.05**
Apnea 2	0.7826	**<0.05**	0.0680	**<0.05**	0.1283	**<0.05**
Apnea 3	0.1876	**<0.05**	0.1331	**<0.05**	0.1062	0.1065
Apnea 4	0.1161	**<0.05**	**<0.05**	**<0.05**	**<0.05**	**<0.05**
Apnea 5	0.7511	**<0.05**	0.6018	**<0.05**	0.0535	0.2354
Tube malposition	0.1661	0.1058	0.0547	**<0.05**	0.7600	0.8454
Pneumothorax	**<0.05**	0.0812	**<0.05**	**<0.05**	0.0936	0.8580
Aspiration	0.2185	0.7474	0.8681	0.1190	0.8555	0.6878

Statistically significant *p*-values are in bold.

Because the registration of the signal occurred with six different measurement coils (Fig. 2), analysis of each measurement coil showed that coil number 4, followed by coil number 2, and coil number 6 were the ones that registered a change in the level of the amplitude after induction of all four pathologies (*p* ≤ 0.05; Table 3). Coil numbers 1 (significant change only in pneumothorax; *p* ≤ 0.05), 3 (significance only in apnea 4 and pneumothorax; *p* ≤ 0.05), and 5 (apnea 1 and 4; *p* ≤ 0.05) were only able to register single pathologies (Table 3).

## Discussion

In our study, we could extend our established newborn animal model with induced pathologies. These pathologies were also registered by standard monitoring with pulse oximetry, ECG, and X-ray.

A significant change in the level of the amplitude of the signal was registered by MIM in all five induced phases of apnea. This also took place after malposition of the tube and induction of the pneumothorax. The induction of a common pathology and respiratory settings were performed according to the study protocol (Table 1). We are therefore confident that every single animal experiment is comparable and all together representative for a typical practical emergency situation in a NICU.

Aspiration alone did not produce a significant change in the level of the amplitude. Although changes could be registered, they were only minor. One reason could be that the isotonic saline solution has a high conductivity and, therefore, a high sensitivity for detection via MIM. Premature and newborn infants were used to aspirate the milk, which has much less conductivity than the saline solution. Fluids with less conductivity could probably be registered better.

We note that aspiration was the last pathology induced and resulted in a significantly lower breathing rate and mechanics (e.g., depth of inspiration) of the experimental animal. This could cause a shift in the breathing mechanics resulting in a more distinctive rise and fall of the amplitude. A few technical problems (coupling and positioning of the measurement coils) also added a partial worsening of the signal quality. This had no significant impact on the measurement proceedings.

The registration quality of the measurement signal differed between the six coils at the bottom of the experimental incubator. While coil numbers 4, 2, and 6 registered a change in the level of the amplitude in all four pathologies (Table 3), the other three coils registered a significant change only in one or, at least, two pathologies. Coil number 3 only registered in apnea 4 and in pneumothorax, while coil numbers 1 and 5 showed a significant change in only one pathology (respectively, pneumothorax and apnea 1 and 4; Table 3).

A possible explanation in addition to the technical problems mentioned above is the positioning of the animals in the tailored foam pad related to the localization of the measurement coils (Fig. 2).

A possible side effect of MIM is a local warming due to electromagnetic radiation,^22^ which could be an important issue for the extremely premature organism and growth potential of preterm infants. Theoretically, the whole energy input is ~200 mW,^20^ which is a factor of 10 below the specific absorption rate of 2 W/kg allowed.^20^ In our previous study, we could show an energy input far below the specific absorption rate maximum allowed.^21^

We were able to register acute respiratory pathologies of premature infants in daily routine care of a NICU through an increase of the sensitivity of the measurement coils compared to further studies.^20,21^

From our current level of experience and understanding, it seems fair to state that MIM is a noncontact monitoring method with a medium subject sensor distance (about the coil diameter, 3–6 cm) that has several advantages: It can penetrate textiles and most coverings (if nonmetallic), does not need a free line of sight (unlike all camera-based methods, which have also attracted a lot of attention recently), and the signal correlates strongly with both local ventilation and—to a lesser extent—local perfusion. However, due to the active induction of (small) eddy currents, MIM is a modality that will need medical approval prior to application in humans, even though simulations have indicated that the thermal load induced inside the body is negligible.

Although our results under experimental conditions suggest some clinical benefit, there are limitations of the method. The aim of our study was a “proof of concept” showing that MIM is able, under some conditions, to detect lung pathologies as long as they affect respiratory mechanics. Unfortunately, aspiration—a common incident in the preterm age—was not detected sufficiently. Additionally, the experimental animals were analgosedated during the whole experiment, a condition related to only a few patients of a NICU. This setup only reflects a small part of possible acute pulmonary problems of premature and mature infants.

The method of magnetic impedance monitoring is based on the near-field electromagnetic induction principle. The penetration depth is about the coil size (3–6 cm), which will be sufficient for the infant thoracic monitoring if the coils can actually be positioned in the mattress. The magnetic impedance (and related emerging impedance-based methods, such as electrical impedance tomography) do not provide absolute impedance measurements but relative changes. Hence, they always require a baseline against which to compare.

The induction of eddy currents is harmless but not yet approved for medical use. Hence, medical-grade approval has to be achieved prior to commercial use.

In summary, MIM seems to be a noncontact method in which ventilation disorders of premature and mature infants can possibly be detected. We emphasize that the position of the measurement coil depending on the position of the experimental animal seems to be especially important. In addition to the possibility of an early detection of acutely developing ventilation problems, perhaps potential information for therapeutical interventions, such as inhalations or medical respiratory analepsis, will become possible via MIM.
